# Estimation of the effect of vaccination in critically ill COVID-19 patients, analysis using propensity score matching

**DOI:** 10.1186/s13613-024-01257-7

**Published:** 2024-02-12

**Authors:** Amarja Ashok Havaldar, Sumithra Selvam

**Affiliations:** 1 Department of Critical Care, St. John’s Medical College Hospital, Bangalore, 560034 India; 2 Department of Biostatistics, St. John’s Research Institute, Bangalore, 560034 India

**Keywords:** Breakthrough infection, COVID-19, ICU, Mortality, Propensity score matching, Unvaccinated, Vaccination

## Abstract

**Background:**

Vaccination helped in reducing mortality and disease severity due to COVID-19. Some patients can develop breakthrough infections. The effect of vaccination in critically ill patients admitted with breakthrough infections is not well studied. We designed a study to estimate the effect of vaccination on ICU mortality in critically ill COVID-19 patients by using propensity score matching.

**Methods:**

We included patients from 15th June 2020 to 31st December 2021. Inclusion criteria were unvaccinated and vaccinated COVID-19 patients requiring intensive care unit (ICU) admission. The institutional ethics committee approval was obtained (institutional ethics committee, IEC 08/2023, Clinical trial registry, India CTRI/2023/01/049142). The primary outcome was ICU mortality. The secondary outcomes were the length of ICU stay and duration of mechanical ventilation. We used multivariable logistic regression (MLR) and propensity score matching (PSM) for the statistical analysis.

**Results:**

Total of 667 patients (79.31%) were unvaccinated and 174 (20.68%) vaccinated. The mean age was 57.11 [standard deviation (SD) 15.13], and 70.27% were males. The ICU mortality was 56.60% [95% confidence interval (CI) 53.24–60%]. The results of MLR and PSM method showed that vaccinated patients were less likely to be associated with mortality [adjusted odds ratio (AOR), 95% CI using logistic regression: 0.52 (0.29, 0.94), and by propensity score matching: 0.83 (0.77, 0.91)].

**Conclusion:**

The findings of this study support COVID-19 vaccination as an effective method for reducing case fatality not only in the general population but also in critically ill patients, and it has important public health implications.

## Introduction

The COVID-19 pandemic has caused varied presentations, primarily affecting the respiratory system leading to pneumonia. The disease had different severity ranging from mild or asymptomatic infection to a severe disease requiring intensive care unit admission [1]. The intensivists used different therapies during the first wave. The use of steroids helped in reducing mortality in patients requiring oxygen therapy [2]. Vaccination was one of the effective measures to contain the pandemic [3, 4]. Breakthrough infections can occur despite vaccination. We defined a breakthrough infection as a COVID-19 infection resulting after the first or second dose of vaccination. The presence of comorbidities and severe COVID-19 infection resulted in the requirement for ICU care. There is a paucity of data on the effect of vaccination in the ICU population.

The population-based study from Israel showed two doses of the BNT162b2 vaccine reduced symptomatic as well as asymptomatic COVID-19 infections, and breakthrough infection was less severe with reduced hospitalisation and lower mortality [5]. A similar study from the Calabria region of Italy on BNT162b2 vaccination showed significantly lesser mortality (24.3%) in the vaccinated as compared to unvaccinated patients (38.5%) [6]. A recent study from Turkey showed BioNTech and Sinovac vaccination reduced the severity of illness, need for invasive ventilation, and mortality. The hospital mortality was 41.5% in vaccinated as against 64.9% in the unvaccinated patients [7]. The UK app-based study from London showed frailty was one of the risk factors associated with breakthrough infections in the above 60 years age group after the first dose. The study participants had received BNT162b2, ChAdOx1nCoV19, or mRNA-1273 vaccine. The symptoms were less frequent in the vaccinated individuals [8].

Grasselli et al., showed vaccination with mRNA or adenoviral vector vaccine reduced ICU admissions for COVID-19 infection. The vaccinated patients were older and had more comorbidities. There was no association between vaccination status and mortality [9]. Similar observations were seen in the studies from Switzerland and Spain [10, 11]. The Australian study showed higher ICU and hospital mortality in vaccinated patients. After adjusting for the covariates, the mortality in the vaccinated and unvaccinated patients was similar [12]. As the results from the available literature are inconsistent, there is a need to know whether vaccination reduced the mortality in critically ill patients who developed breakthrough infections after vaccination. We aimed to study the effectiveness of vaccination in critically ill COVID-19 patients who developed breakthrough infection by propensity score matching.

## Methods

The institutional ethical committee approval was obtained, IEC/08/2023, CTRI/2023/01/049142, and data were retrieved. Patients were included from 15th June 2020 to 31st December 2021. The current study includes data from 2 multicentre published studies [13, 14], consisting of 841 patients. The epidemiology and ventilation characteristics of confirmed cases of severe COVID-19 pneumonia admitted in intensive care unit (EPIC19) study described the epidemiology and ventilation characteristics of the unvaccinated COVID-19 patients [13]. The second study, the Postcovac-covid group, described the characteristics of the patients who developed breakthrough infections after COVID-19 vaccination. The patients of the Postcovac-covid study had received either ChAdOx1 nCov19 (Covishield) or BBV 152 COVID-19 (Covaxin) vaccine. The median time from vaccination to the hospitalisation was 33.5 days [14]. The baseline characteristics like age, gender, comorbidities, arterial blood gas (ABG) pH, and Pao2/Fio2 ratio (PF ratio) were collected. The ABG pH corresponds to the worst pH value during the first 24 h of ICU admission. The PF ratio defined as the ratio of partial pressure of oxygen (PaO2) divided by fraction of inspired oxygen (Fio2).

The acute physiology, age, and chronic health evaluation (APACHE II) score and sequential organ failure score (SOFA) were collected. The APACHE II considers various laboratory and clinical parameters and the presence of acute and chronic diseases [15]. It provides information about the severity of the illness and estimates the mortality. The SOFA score evaluates organ failure involving six organ systems and estimates the mortality [15, 16]. The primary outcome was the effect of vaccination on ICU mortality. The secondary outcomes were the length of ICU stay and duration of mechanical ventilation.

### Statistical analysis

Continuous variables represented as mean (standard deviation SD) for the normally distributed variables or median with 25th and 75th percentiles for non-normal variables. The categorical variables presented as numbers with percentages. Initial analyses were performed using independent t-test and Mann–Whitney *U* test as applicable, to compare the clinical characteristics such as age, APACHE II score, SOFA score, ABG pH, and PF ratio, duration of mechanical ventilation, and length of ICU stay between vaccinated and unvaccinated patients. The association between categorical variables and vaccination status was assessed using the chi-squared test. The significant variables in the univariate analysis and clinically relevant were considered for multivariable logistic regression analysis (MLR). The MLR was performed to compare the ICU mortality between vaccinated and unvaccinated patients adjusted for covariates. Propensity score matching (PSM) was performed.

### Propensity score matching (PSM)

PSM is to estimate the effect of treatment when randomisation is not possible. In randomised controlled trials, random assignment of patients into intervention and control groups balances individuals for all the observed and unobserved characteristics. Whereas in observational studies, the treatment assignment is not random and causes an imbalance in the baseline characteristics, leading to a selection bias. PSM is the recommended statistical method for balancing the measured covariates between treated and control groups.

The propensity score is a balancing score. In PSM, treatment and control patients are paired based on similar propensity scores and possibly other covariates. It is the probability of treatment assignment conditional on the observed baseline covariates, e_*k*_ = *Pr(Z*_*k*_ = 1|X_*k*_). In this equation, for subject k, *Z*_k_ = 1 is the treatment assigned, and *X*_k_ is the vector of observed covariates. We had a higher number of unvaccinated than vaccinated patients; hence, we used the matching procedure of PSM as the recommended method [17–19]. The vaccinated and unvaccinated patients were matched on the estimated propensity score. For PSM, covariates that are potentially related to the outcome were included. The covariates selected were age, gender, comorbidities, APACHE II score, need for invasive ventilation, ABG pH, need for renal replacement therapy, and use of steroids.

After performing the PSM using matching method, a check for the balance of individual covariates across vaccinated and unvaccinated patients for the estimated propensity score was performed using a Kernel density plot. Each vaccinated individual was assigned a weight of one. In order to match the individuals between vaccinated and unvaccinated groups, weighted composite of comparison observations was considered. The comparison patient’s propensity scores were weighted within a range of propensity scores according to the distance, and they were from the vaccinated subjects [18]. The observations outside of the common support range and showed no overlap between vaccinated and unvaccinated patients were excluded. Out of 841 patients, 718 patients were in the range of common support, hence included in the further analysis. The Kernel density plot before matching and after matching of the covariates was plotted for vaccinated and unvaccinated patients. The effect of vaccination was estimated and reported using the average treatment effect (ATE) for the entire sample. The ATE is estimated from a sample using a comparison in mean outcomes for treated and untreated units. The statistical analysis was performed using STATA™ (Version 14, College Station TX) software. The *p* value < 0.05 was considered as statistically significant.

## Results

A comparison of baseline characteristics between vaccinated and unvaccinated groups is presented in Table 1. We included 841 patients in the analysis, 667 (79.31%) unvaccinated and 174 (20.68%) vaccinated patients (Table 1).

**Table 1:** Baseline characteristics in the vaccinated and non-vaccinated population

Parameters	All (841)	Vaccinated (174)	Unvaccinated (667)	*p* value
Age in years^a^	57.11 ± 15.13	57.54 ± 14.60	57.00 ± 15.28	0.676
Gender (male/female)	591/250 (70.27/29.73)	121/53 (69.54/30.46)	470/197 (70.46/29.54)	0.852
Diabetes mellitus	445(52.91)	70(40.23)	375(56.22)	< 0.001
Hypertension	443(52.68)	62(35.63)	381(57.12)	< 0.001
Chronic kidney disease	100(11.89)	10(5.75)	90(13.49)	0.005
Chronic liver disease	20(2.38)	5(2.87)	15(2.25)	0.630
Ischaemic heart disease	116(13.79)	18(10.34)	98(14.69)	0.139
Immunosuppressants	28(3.33)	2(1.15)	26(3.90)	0.072
Malignancy	16(1.90)	4(2.30)	12(1.80)	0.667
COPD	31(3.69)	4(2.30)	27(4.05)	0.275
Bronchial asthma	30(3.57)	5(2.87)	25(3.75)	0.580
Interstitial lung disease	7(0.83)	2(1.15)	5(0.75)	0.605
Retroviral disease	2(0.24)	0(0)	2(0.30)	0.470
Tuberculosis	17(2.02)	2(1.15)	15(2.25)	0.359
APACHE II score^b^	28(23–33)	14(8–24.50)	29(26–34)	< 0.001
SOFA score^b^	7(4–10)	6(4–8)	7(4–11)	0.005
Invasive mechanical ventilation	350(41.62)	46(26.44)	304(45.58)	< 0.001
Need for RRT	92(11.65)	8(6.50)	84(12.59)	0.053
ABG pH^a^	7.34 ± 0.13	7.38 ± 0.11	7.33 ± 0.13	< 0.001
Steroid usage	719(85.49)	123(70.69)	596(89.36)	< 0.001
PaO2/FiO2 ratio^b^	110 (78.35–182.5)	113.33 (82.27–175)	109.31 (77.79–187.26)	0.724
Duration of mechanical ventilation^b^ (In days)	7(3–11)	7.5(4.5–13)	6(3–11)	0.158
Length of ICU stay^b^ (In days)	7(4–13)	8(4–14)	7(4–13)	0.442

Reported as number (%), ^a^Mean ± SD, ^b^Median (25th, 75th percentile)

The mean age was 57.11 (SD 15.13), and predominantly male patients (70.27%). The mean age was comparable between the groups (*p =* 0.676). There was no effect of gender on the vaccination status (*p =* 0.852). The unvaccinated group had a significantly higher proportion of diabetes mellitus, hypertension, and chronic kidney disease as compared to the vaccinated group (*p <* 0.001). The APACHE II and SOFA scores were significantly higher in the unvaccinated group (*p <* 0.01). The invasive ventilation support requirement was significantly higher in the unvaccinated group (45.58%) compared to the vaccinated group (26.44%) (*p <* 0.001). The mean ABG pH was significantly lower in unvaccinated patients (*p <* 0.001). The PF ratio was similar between the vaccinated and unvaccinated groups. However, the use of steroids was significantly higher in unvaccinated patients. The median PF ratio was significantly lower in patients on steroids [108 (77–175)] as compared to patients not on steroids [160 (94, 268)] (*p <* 0.01).

### Primary outcome

The ICU mortality was 56.60% (95% CI 53.24–60%). The proportion of mortality was significantly lower among vaccinated patients than unvaccinated patients (43.7% Vs 60.0%, *p <* 0.0001). The results of multivariable logistic regression and propensity score matching are presented in Table 2.

**Table 2:** Comparison of the results of MLR and PSM analysis for ICU mortality

Parameters	MLR AOR 95% CI	*p* value	PSM AOR 95% C.I	*p* value
Vaccinated	0.52 (0.29, 0.94)	0.03	0.83 (0.77, 0.91)	< 0.001
Age (in years)	1.02(1.004,1.03)	0.010		
Gender	1.05 (0.73, 1.50)	0.80		
Diabetes mellitus	1.08 (0.74, 1.56)	0.69		
Hypertension	0.94 (0.63, 1.38)	0.78		
APACHE II score	1.07 (1.03, 1.10)	< 0.001		
Invasive mechanical ventilation	2.91 (1.95, 4.35)	< 0.001		
Need for RRT	0.93 (0.52, 1.65)	0.91		
ABG pH	0.31 (0.06, 1.67)	0.17		
Steroid usage	2.02 (1.14, 3.58)	0.015		

MLR, Multivariable logistic regression; PSM, Propensity Score Matching; AOR, Adjusted Odds Ratio; CI, Confidence interval (lower limit and upper limit)

**Fig. 1:**
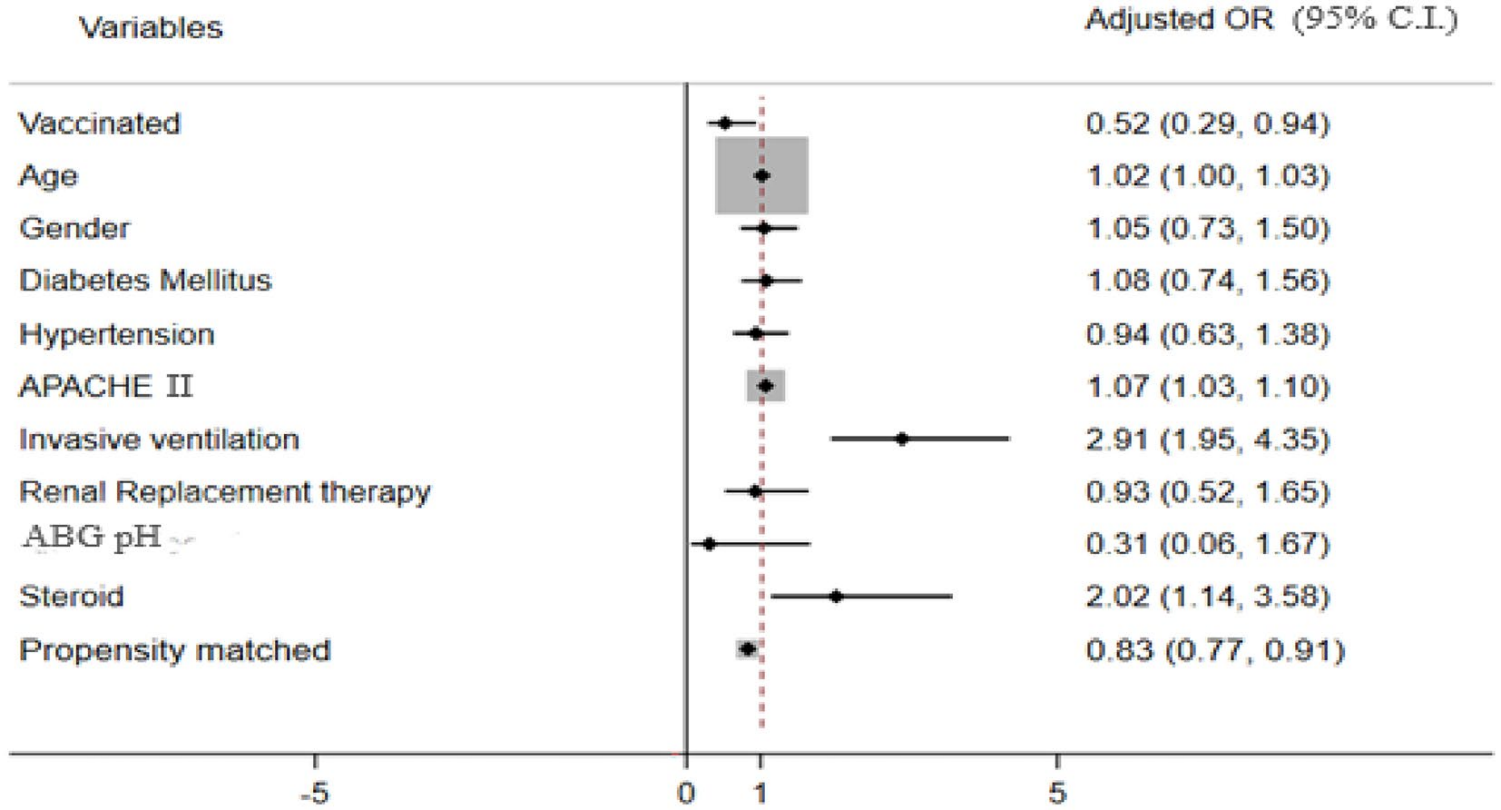
Forest plot of the results MLR and PSM showing adjusted OR with 95% C.I for the ICU mortality. Reported values are adjusted odds ratio (AOR), AOR below 1 indicates the decreased odds of mortality, and AOR above 1 indicates the increased odds of mortality.  Vertical line indicates OR of 1- no significant difference between vaccinated and unvaccinated group. ♦ indicates adjusted odds ratio for each covariate.  Horizontal line indicates lower and upper 95% confidence interval for the adjusted odds ratio for each covariate

The results of logistic regression showed that vaccinated patients had significantly lesser odds of mortality [adjusted odds ratio (AOR), 95% CI 0.52 (0.29, 0.94), *p =* 0.03] compared to the unvaccinated group adjusted for age, gender, diabetes mellitus, hypertension, APACHE II score, invasive ventilation, ABG pH, need for renal replacement therapy (RRT), and use of steroids (Table 2). The MLR showed higher odds of ICU mortality for age (AOR 1.02, 95% CI 1.004–1.03, *p =* 0.010), APACHE II score (AOR 1.07, 95% CI 1.03–1.10, *p <* 0.001), need for invasive ventilation (AOR 2.91, 95% CI 1.95–4.35, *p <* 0.001), and use of steroids (AOR 2.02, 95% CI 1.14–3.58, *p =* 0.015). Estimation of vaccination effect by ATE method of PSM analysis also showed that vaccinated patients had significantly lesser odds of mortality compared to the unvaccinated patients (AOR, 95% CI using PSM: 0.83 (0.77, 0.91), *p <* 0.001). Figure 1 depicts the results of the MLR and PSM using a forest plot explaining the pictorial representation of decreased odds of ICU mortality among vaccinated patients.

**Fig. 2:**
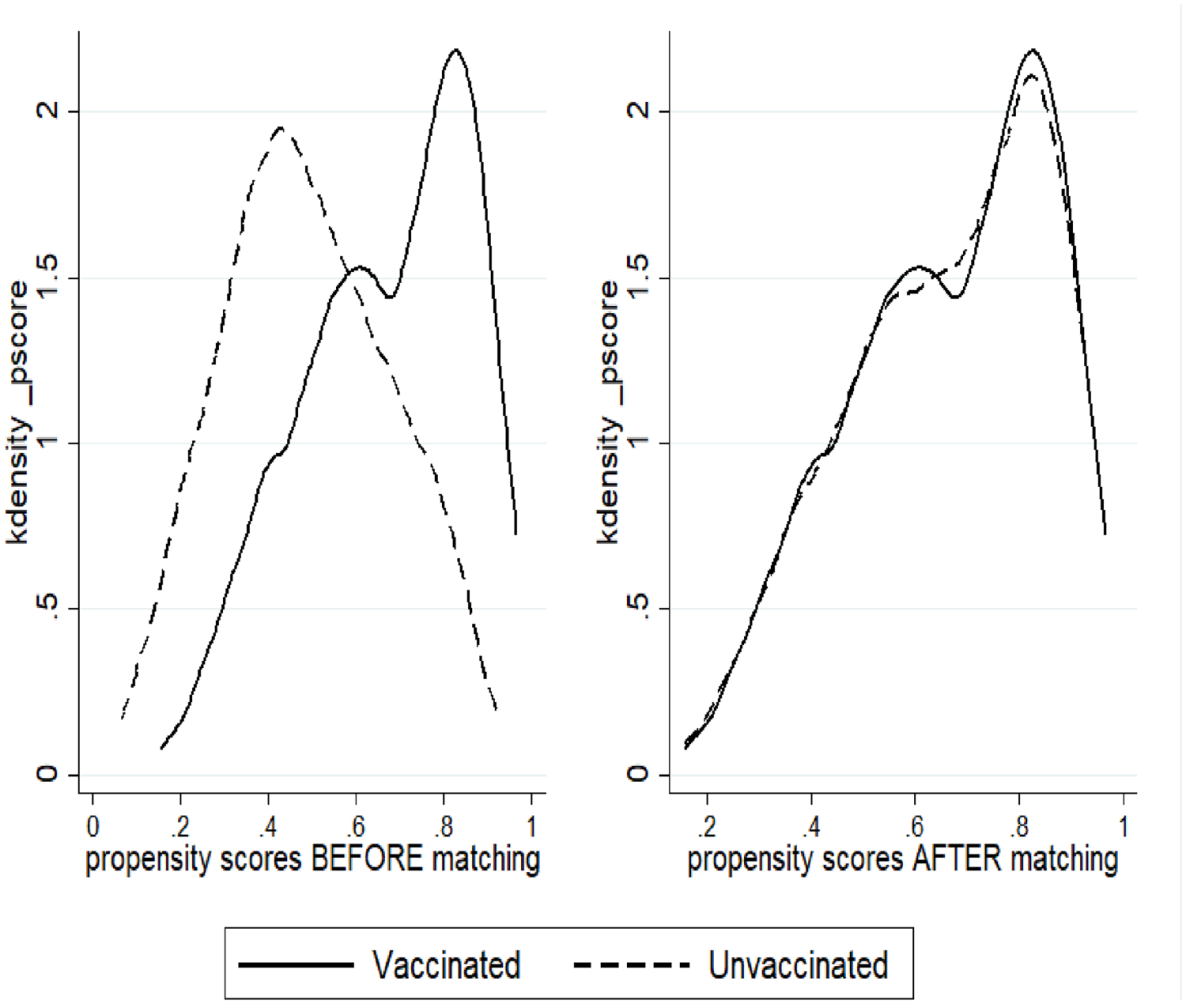
Kernel density plot of the propensity scores before and after matching. kdensity_pscore—Kernel density propensity scores

While comparing the estimations of the PSM results with the conventional logistic regression method, the PSM had a lower standard error with a narrower confidence interval than MLR, indicating the estimation of the average treatment effect using PSM was robust. The probability density scores of the vaccinated and the unvaccinated groups before and after the PSM matching for baseline covariates using Kernel density plots are presented in Fig. 2. The Kernel density plots were similar between the vaccinated and unvaccinated groups after matching for baseline covariates using PSM, indicating a strong balance on the propensity scores among the matched samples.

### Secondary outcomes

Secondary outcomes presented in Table 1. The overall median duration of mechanical ventilation and length of ICU stay were 7 days and were comparable between the vaccinated and unvaccinated groups.

## Discussion

This study showed vaccination reduced ICU mortality. The estimate of the effect of vaccination by PSM had a lower standard error and a narrower confidence interval than shown by MLR. The vaccinated patients had less severe disease as shown by lower median APACHE II score, normal ABG pH, less requirement of invasive ventilator support, and steroids. There was no difference in secondary outcomes among vaccinated and unvaccinated patients.

A study from Calabria, Italy, showed lower mortality in vaccinated patients than unvaccinated patients (24.3% vs. 38.5%, *p =* 0.014) [6]. The vaccinated patients were elderly with a mean age of 67 years (SD 11) and had a significantly higher proportion of comorbidities such as chronic renal failure, autoimmune diseases, and malignancy. Vaccinated patients had a higher PF ratio and lower requirement of invasive ventilator support, but there was no difference in ABG pH among vaccinated and unvaccinated groups. The current study showed no difference in the mean age among vaccinated and unvaccinated groups. The unvaccinated group had higher APACHE II score, increased requirement for invasive ventilator support, steroids, and had a lower ABG pH. This indicates more severe disease in the unvaccinated patients causing alteration in normal physiology and lower ABG pH. The mortality was significantly higher in unvaccinated patients than vaccinated patients (60% vs 43.7%, *p <* 0.0001). Similar mortality was observed in a study from Turkey [7].

A Spanish study showed vaccinated patients had higher comorbidities and higher APACHE II score 12 (9–17) than unvaccinated patients. There was no difference in the ICU mortality among vaccinated (33.3%) and unvaccinated (28.6%) patients (*p =* 0.52) [11]. The unvaccinated patients had a higher length of ICU stay than vaccinated patients, but the duration of invasive mechanical ventilation was not different. The vaccinated patients in our study had lower comorbid illnesses and lower median APCHE II score of 14 (8–24.5) than unvaccinated patients. In comparison with the Spanish study, the median APACHE II score in vaccinated patients was higher in our study. We did not find any difference in the length of ICU stay and duration of mechanical ventilation in vaccinated and unvaccinated groups.

The cross-sectional study from Australia by Madeleine Otto, et al. describes the characteristics of vaccinated and unvaccinated patients. The vaccinated patients in the Australian study had higher ICU mortality than unvaccinated patients (18.3% vs 14, *p <* 0.005). The difference in mortality could be due to elderly population with median age of 64 (51–73) years. The reason stated by the author was the immune escape phenomenon resulting in reduced production of anti-spike antibodies. However, adjusted analysis did not show any difference in mortality between the vaccinated and unvaccinated groups [12].

French study evaluating the impact of vaccination on the severity of illness also showed that the vaccinated cohort was older than the unvaccinated (75 vs.55 yrs) and had a higher proportion of comorbid diseases than the unvaccinated cohort [20]. Although the intensive care unit admissions were lesser in the vaccinated group, the mortality was comparable between the vaccinated and unvaccinated patients in the French study [20]. A study by Grasselli et al., also showed no difference in mortality among vaccinated and unvaccinated patients [9].

Suleyman et al., showed vaccinated patients with breakthrough infection had reduced mortality (11%) as compared to unvaccinated patients (24.9%) (*p <* 0.001), although the severity of illness was similar between study participants based on the modified SOFA score [21]. Lower mortality observed in the study could be due to inclusion of all the patients requiring hospitalisation. However, in our study the effect of vaccination was estimated among ICU population. We noted significantly higher median SOFA score 7 (4–11) in the unvaccinated patients (*p =* 0.005).

The strength of this study is this is the first study which compared the mortality in vaccinated and unvaccinated ICU patients by matching the baseline covariates. We suggest using propensity matching to compare the effect of vaccination on ICU mortality. The important observation is vaccination can help in reducing mortality even in the critically ill population.

The limitation of this study is the information on anti-spike antibody titres in the vaccinated group was unavailable. Hence, we could not study the effect of antibody titres on mortality. Also, data regarding genome sequencing to determine the strain causing the infection were unavailable. There is a lot of heterogeneity observed in various studies describing breakthrough infections. One reason could be that different types of vaccines with varying effectiveness could have affected mortality. Also, the time from vaccination can influence the development of breakthrough infections and mortality. The early breakthrough infection could be due to inadequate immune response, and delayed infection could be due to waning immunity [22]. Although vaccination has shown a reduction in mortality in our study, the response of each patient to control COVID-19 infection will be variable. Future research should focus on studying the host immune response to a particular infection as it is one of the parameters in the epidemiological triad.

Conclusion- Estimation of the effect of vaccination by PSM showed lower mortality in the vaccinated COVID-19 patients admitted to ICU. Older age, higher APACHE II score, need for invasive ventilation, and use of steroids had higher odds for ICU mortality. Vaccination is one of the effective tools in controlling the pandemic and has important public health implications.

## Data Availability

Data are available on a reasonable request.

## Abbreviations

ABG: Arterial blood gas
AOR: Adjusted odds ratio
APACHE II: Acute physiology, age, and chronic health evaluation
ATE: Average treatment effect
CI: Confidence Interval
CTRI: Clinical trial registry India
ICU: Intensive care unit
IEC: Institutional ethics committee
mRNA: Messenger Ribonucleic acid
MLR: Multivariable logistic regression
PSM: Propensity score matching
RRT: Renal replacement therapy
SD: Standard Deviation
SOFA: Sequential organ failure assessment

## References

[1] Zhu N, Zhang D, Wang W, Li X, Yang B, Song J (2020). A novel coronavirus from patients with pneumonia in China, 2019. N Engl J Med.

[2] Horby P, Lim WS, Emberson JR, Mafham M, Bell JL, RECOVERY Collaborative Group (2021). Dexamethasone in hospitalized patients with COVID-19. N Engl J Med.

[3] https://www.who.int/news-room/feature-stories/detail/vaccine-efficacy-effectiveness-and-protection. Assessed 11 Jan 2022.

[4] Onder G, Rezza G, Brusaferro S (2020). Case-fatality rate and characteristics of patients dying in relation to COVID-19 in Italy. JAMA.

[5] Haas EJ, Angulo FJ, McLaughlin JM, Anis E, Singer SR, Khan F (2021). Impact and effectiveness of mRNA BNT162b2 vaccine against SARS-CoV-2 infections and COVID-19 cases, hospitalisations, and deaths following a nationwide vaccination campaign in Israel: an observational study using national surveillance data. Lancet.

[6] Bruni A, Longhini F, Macheda S, Biamonte E, Pasqua P, Neri G, Guzzo ML, Garofalo E, Caroleo A, Chirico P, Corea A (2022). Characteristics of unvaccinated and vaccinated critically ill COVID-19 patients in calabria region (Italy): a retrospective study. Front Med.

[7] Koc I, Ozmen SU, Deniz O (2023). Vaccine effectiveness against the B. 1.617. 2 in the intensive care unit. Medicine.

[8] Antonelli M, Penfold RS, Merino J, Sudre CH, Molteni E, Berry S (2022). Risk factors and disease profile of post-vaccination SARS-CoV-2 infection in UK users of the COVID Symptom Study app: a prospective, community-based, nested, case-control study. Lancet Infect Dis.

[9] Grasselli G, Zanella A, Carlesso E, Florio G, Canakoglu A, Bellani G, Bottino N, Cabrini L, Castelli GP, Catena E, Cecconi M (2022). Association of COVID-19 vaccinations with intensive care unit admissions and outcome of critically ill patients with COVID-19 pneumonia in Lombardy, Italy. JAMA Netw Open.

[10] Hilty MP, Keiser S, Wendel Garcia PD, Moser A, Schuepbach RA (2022). mRNA-based SARS-CoV-2 vaccination is associated with reduced ICU admission rate and disease severity in critically ill COVID-19 patients treated in Switzerland. Intensive Care Med.

[11] Motos A, López-Gavín A, Riera J, Ceccato A, Fernández-Barat L, Bermejo-Martin JF, Ferrer R, de Gonzalo-Calvo D, Menéndez R, Pérez-Arnal R, García-Gasulla D (2022). Higher frequency of comorbidities in fully vaccinated patients admitted to the ICU due to severe COVID-19: a prospective, multicentre, observational study. Eur Respir J.

[12] Otto M, Burrell AJ, Neto AS, Alliegro PV, Trapani T, Cheng A, Udy AA (2023). Clinical characteristics and outcomes of critically ill patients with one, two and three doses of vaccination against COVID-19 in Australia. Intern Med J.

[13] Havaldar AA, Kumar MV, Vijayan B, Prakash J, Kartik M, Sangale A (2022). Epidemiology and ventilation characteristics of confirmed cases of severe COVID-19 pneumonia admitted in intensive care unit (EPIC19): a multicentre observational study. Indian J Anaesth.

[14] Havaldar AA, Prakash J, Kumar S, Sheshala K, Chennabasappa A, Thomas RR (2022). Demographics and clinical characteristics of COVID-19-vaccinated patients admitted To ICU: a multicenter cohort study from India (PostCoVac study-COVID group). Indian J Crit Care Med.

[15] Knaus WA, Draper EA, Wagner DP, Zimmerman JE (1985). APACHE II: a severity of disease classification system. Crit Care Med.

[16] Vincent JL, Moreno R (2010). Clinical review: scoring systems in the critically ill. Crit Care.

[17] Thavaneswaran A, Lix L (2008). Propensity score matching in observational studies.

[18] Garrido MM, Kelley AS, Paris J, Roza K, Meier DE, Morrison RS, Aldridge MD (2014). Methods for constructing and assessing propensity scores. Health Serv Res.

[19] Austin PC (2011). An introduction to propensity score methods for reducing the effects of confounding in observational studies. Multivar Behav Res.

[20] Epaulard O, Abgrall S, Lefebvre M, Faucher JF, Michon J, Frentiu E, Blanchi S, Janssen C, Charbonnier G, Fresse A, Laurent S (2022). Impact of vaccination on the symptoms of hospitalised patients with SARS-CoV-2 delta variant (B. 1.617. 1) infection. Clin Microbiol Infect.

[21] Suleyman G, Fadel R, Alsaadi A, Sueng LN, Ghandour A, Alkhatib A, Singh T, Parsons A, Miller J, Ramesh M, Brar I (2022). Progression to critical illness and death in patients with breakthrough hospitalizations. Open Forum Infect Dis.

[22] Goldberg Y, Mandel M, Bar-On YM, Bodenheimer O, Freedman L, Haas EJ, Milo R, Alroy-Preis S, Ash N, Huppert A (2021). Waning immunity after the BNT162b2 vaccine in Israel. N Engl J Med.

